# A Novel Muco-Gingival Approach for Immediate Implant Placement to Obtain Soft- and Hard-Tissue Augmentation

**DOI:** 10.3390/jcm11174985

**Published:** 2022-08-25

**Authors:** Martina Stefanini, Matteo Sangiorgi, Diego Bianchelli, Pietro Bellone, Federico Gelpi, Daniele De Santis, Giovanni Zucchelli

**Affiliations:** 1 Department of Biomedical and Neuromotor Sciences, Periodontology Unit, University of Bologna, 40125 Bologna, Italy; 2 Head and Neck Department, Department of Surgery, Dentistry, Pediatrics and Gynecology, University of Verona, 37124 Verona, Italy

**Keywords:** immediate implant placement, muco-gingival approach, soft-tissue augmentation, hard-tissue augmentation, connective-tissue graft

## Abstract

The aim of this article is to describe a novel approach combining muco-gingival, regenerative and prosthetics concepts for immediate implant insertion that overcomes the limits traditionally considered as contraindications for Type 1 flapless implant positioning, simultaneously obtaining soft- and hard-tissue augmentation. After pre-surgical CBCT evaluation, the surgical technique consisted in the execution of a lateral-approach coronally advanced envelope flap, with oblique submarginal interproximal incisions directed towards the flap’s center of rotation (the tooth to be extracted); after buccal-flap elevation, the atraumatic extraction of the tooth was performed. Following guided implant insertion, a mixture of biomaterial and autologous bone was placed, stabilized by a pericardium membrane and a connective-tissue graft sutured in the inner aspect of the buccal flap. The peri-implant soft tissues were conditioned with a provisional crown until the shape and position for the mucosal scallop to resemble the gingival margin of the adjacent corresponding tooth were obtained; then, the definitive screw-retained restoration was placed. Within the limitations of this case report, the proposed immediate implant placement approach combining CTG application and buccal bone regeneration showed the possibility of obtaining 1-year-follow-up implant success, stable bone level, good esthetic results and high patient satisfaction.

## 1. Introduction

Implant placement into the socket during the same surgical session as tooth removal can be classified as immediate [1]. While minimizing the duration of edentulism and the number of surgical interventions may be advantageous for surgeons and patients [2,3], immediate implant placement (IIP) is not able to mitigate buccal hard- and soft-tissue remodeling following tooth extraction [4,5,6,7,8].

This approach is described as Type 1 and is considered a complex procedure according to the SAC Classification (straightforward (S), advanced (A) and complex (C)) [9]; therefore, it should only be applied by talented, well-educated and experienced implant surgeons with proper treatment planning and meticulous execution [10]. However, a recent literature review evaluating immediate implant placement and immediate restoration in the anterior maxilla reported 626 implants with a success rate of 97.96% and a survival rate of 98.25% (medium follow-up: 31.2 months) [11] in accordance with the systematic review of the literature by Del Fabbro et al. [12], who reported an overall implant survival rate of 97.62% (range: 78.6–100%) after 1 year of function. The most common complication following IIP appears to be mid-facial recession [13,14,15] leading to unsatisfactory results, as aesthetic single-tooth replacement encompasses both a natural appearance of the restoration and of the peri-implant mucosa.

Therefore, despite the survival rate of post-extraction implants in the esthetic area is high, there is also a very high risk of mucosal recession, which recommends a careful case selection. The ITI (International Team for Implantology) stated that IIP Type 1 is considered the treatment of choice as a flapless procedure in sites with ideal anatomical conditions, such as an intact facial bone wall with a thick wall phenotype (>1 mm) and a thick gingival biotype [9]. Under these strict selection criteria, this may represent 5–10% of single-tooth extractions in the esthetic zone. For patients, this approach is attractive, since it offers the possibility of an immediate provisional prosthesis being delivered on the day of extraction [16].

The aim of this article is to describe a novel approach combining mucogingival, regenerative and prosthetics concepts for immediate implant insertion that overcomes the aforementioned limits traditionally considered as contraindications for Type 1 flapless implant positioning.

## 2. Case Report

**Figure 1:**
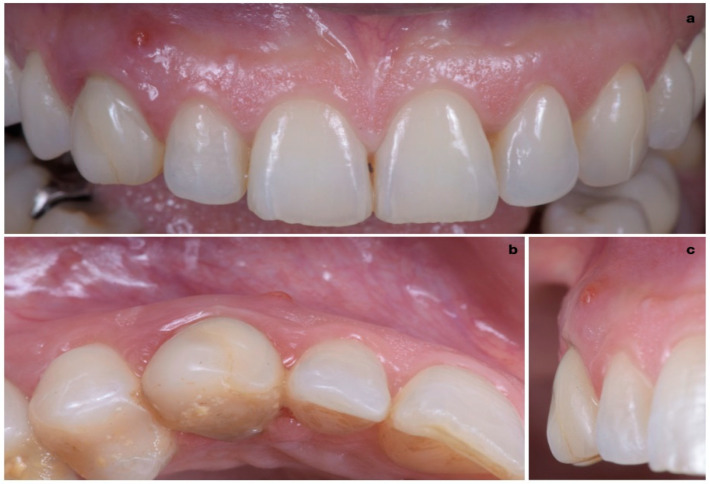
(**a**–**c**) Forty-year-old patient with endodontic fistula on maxillary right canine.

**Figure 2:**
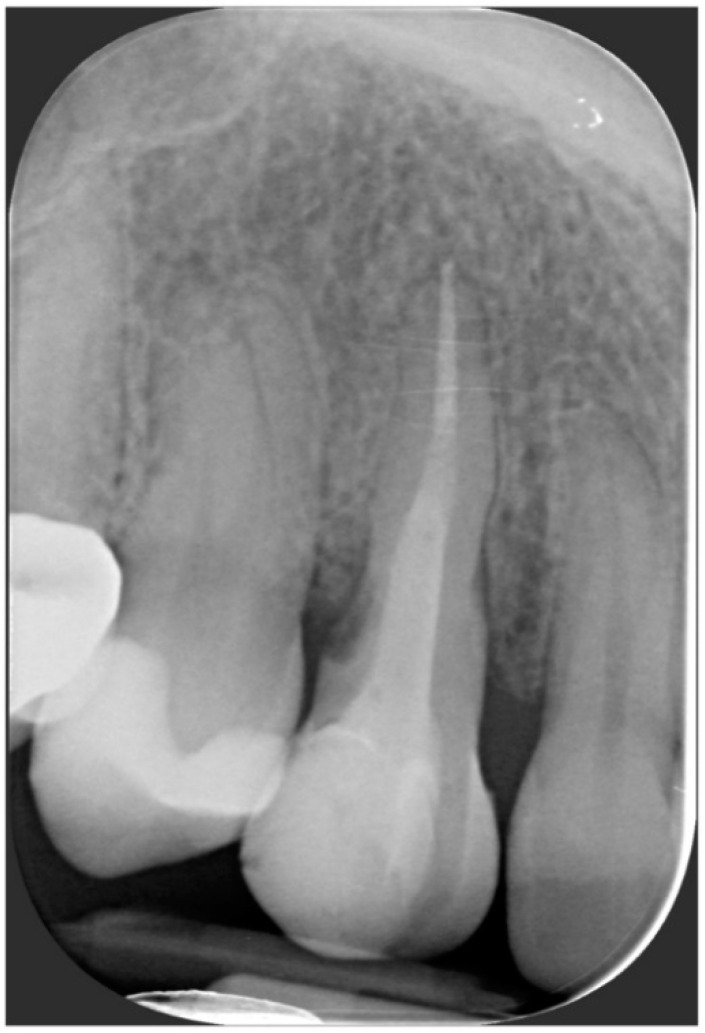
Radiographic evaluation revealing internal root resorption.

A 40-year-old patient presented with an endodontic fistula on the maxillary right canine (Figure 1a–c). Radiographic evaluation revealed internal root resorption (Figure 2). The medical history did not include any systemic conditions contraindicating implant placement or regenerative surgery. The patient did not suffer from any form of periodontal disease. Due to the extension of root resorption, the tooth was considered irrational to treat, and the treatment plan consisted of extraction and immediate implant positioning.

### 2.1. Pre-Surgical Evaluation

Prior to the surgery, CBCT was requested in order to plan a fully guided implant insertion.

The mean axis of the implant coincided with the most palatal extension of the cuspid of the canine of the predetermined final restoration. During planning, it was decided to use a Prama implant with a short neck of 1.8 mm, a diameter of 3.8 mm and a length of 11.5 mm. 

The apical-coronal planning of implant placement was chosen in relation to the esthetically ideal position of the buccal soft-tissue margin of the final restoration; the smooth implant collar was 2 mm apical with respect to the ideal position of the buccal soft-tissue margin of the final restoration. In fact, in an apico-coronal direction, the endosseous implant portion is positioned 3.5–4 mm apical with respect to an imaginary horizontal line that is placed at the same level of the gingival margin of the natural homologous tooth, which always represents the reference point for implant positioning. The use of transmucosal implants (in which the height of the polished neck can be chosen in relation to the peri-implant transmucosal portion) is useful for displacing the implant–abutment connection away from the bone crest. This reduces bone resorption and makes hygiene maintenance easier both for the patient and the hygienist.

**Figure 3:**
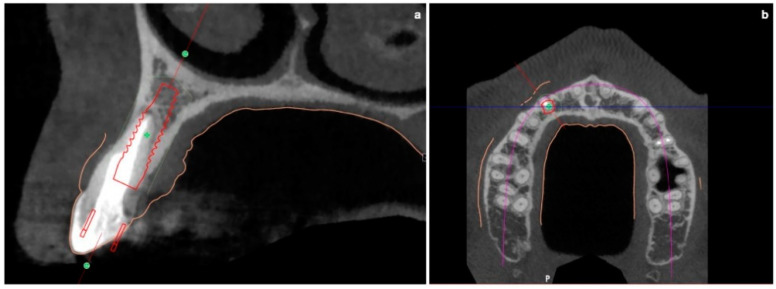
(**a**,**b**) Pre-surgical CBCT evaluation in order to perform guided implant insertion.

The buccal bone wall was extremely thin but partially maintained with 2 mm of buccal bone dehiscence (Figure 3a,b).

### 2.2. Surgical Procedure

**Figure 4:**
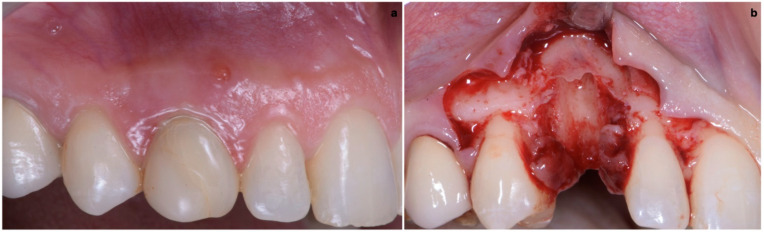
(**a**,**b**) Lateral-approach coronally advanced envelope flap, with oblique submarginal interproximal incisions directed towards the flap’s center of rotation (the tooth to be extracted).

**Figure 5:**
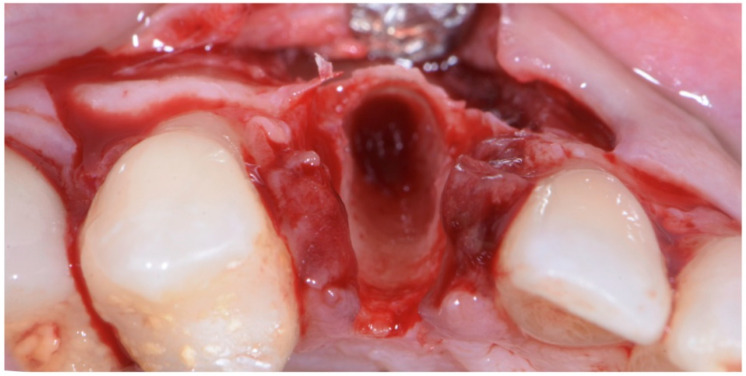
Atraumatic extraction of canine after buccal-flap elevation.

The surgical technique consisted in the execution of a lateral-approach coronally advanced envelope flap, which requires oblique submarginal interproximal incisions directed towards the flap’s center of rotation (in this case, the tooth to be extracted) (Figure 4a,b); the latter start from the gingival margin of the adjacent tooth and end at a distance from the vertex of the papilla that should equal the desired amount of coronal advancement of the marginal tissues. The flap was elevated in a split–full–split manner; a split-thickness incision was performed at the level of the surgical papillae by keeping the blade parallel to the bone; then, the central portion of the buccal flap was elevated in full thickness with a periosteal elevator until 2–3 mm of buccal bone was exposed. Apically, flap elevation continued, first with a “deep” split-thickness incision, keeping the blade parallel to the osseous plane and detaching the muscle insertions from the periosteum, and afterwards with a “superficial” one, placing the blade parallel to the external mucosal surface and controlling its movement using transparency while detaching the superficial muscle insertions from the inner aspect of the flap. This last incision is the one responsible for flap mobility [17]. Once the buccal flap was elevated, the atraumatic extraction of the canine was performed (Figure 5). Full visibility and access help to avoid both trauma to the anatomical papillae and damage to the buccal bone plate; special attention should be placed towards preserving the integrity of the anatomical papillae mesial and distal with respect to the canine, since they are essential for the surgery’s successful outcome.

**Figure 6:**
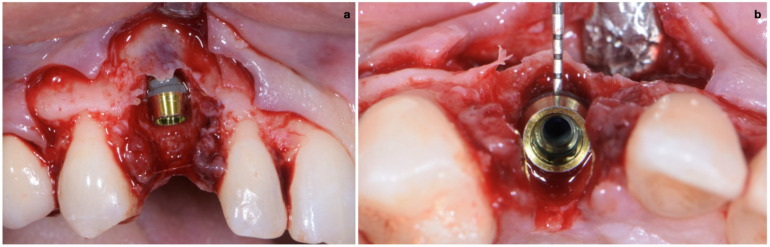
(**a**,**b**) Implant placement after guided preparation of implant site.

The implant site was prepared in a fully guided manner, using drills of progressively larger diameter, so that at the moment of guided implant insertion, the fixture could reach the pre-established position with enough primary stability to consent immediate provisionalization (35 Ncm) (Figure 6a,b). The implant shoulder should be palatal with respect to the imaginary line that connects the buccal profile of the adjacent teeth without placing it so that it is excessively palatal, in order to avoid creating prosthetic emergence profiles that are too horizontal, which are difficult to maintain in terms of hygiene.

**Figure 7:**
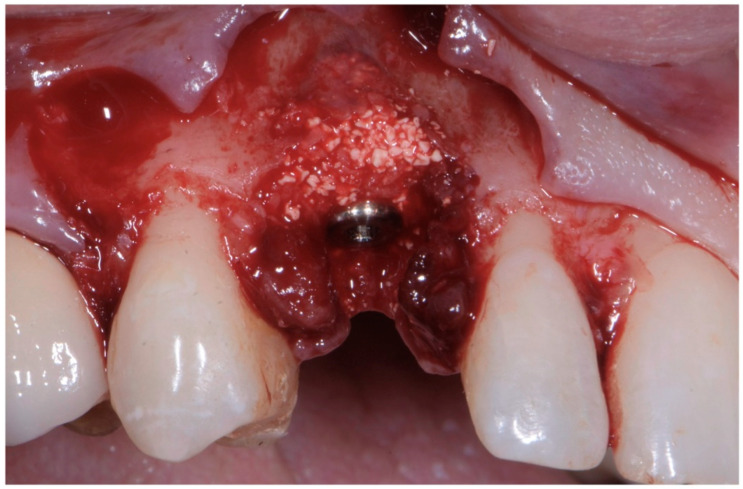
Biomaterial and autologous bone placed to cover the exposed implant threads and to fill the gap between implant surface and socket wall.

**Figure 8:**
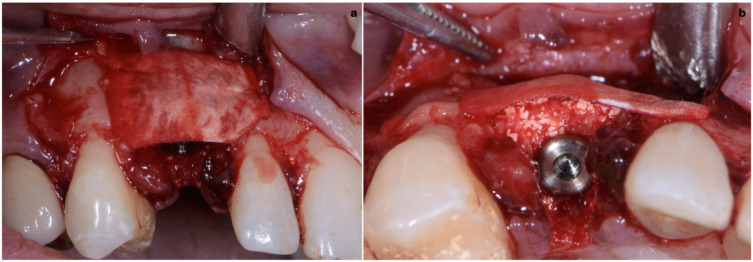
(**a**,**b**) Biomaterial stabilized by a thin pericardium membrane placed between bone particles and connective-tissue graft.

As already evaluated during software planning, after implant insertion, a buccal bone dehiscence of 2 mm occurred. The remaining buccal bone thickness was less than 1 mm. A mixture of biomaterial (*Cerabone; Botiss*) and autologous bone was placed to cover the exposed implant threads and the thin layer of remaining buccal bone and to fill the gap between the implant surface and the socket wall (Figure 7). Bone filler material should be limited to the rough implant surface, extending as little as possible to the transmucosal collar; however, the biomaterial positioning should be slightly in excess to avoid the loss of biomaterial during the following phase. If there is still an excess, it should be removed just before the very last suture. The biomaterial was then stabilized using a thin pericardium membrane (*Jason Membrane; Botiss*) placed between the bone particles and a connective-tissue graft (Figure 8a,b). The pericardium membrane was sutured to the periosteum left appositively laterally to the membrane.

**Figure 9:**
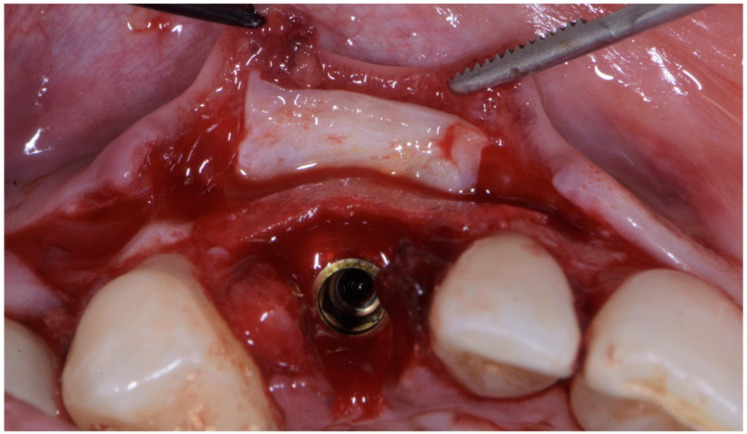
Connective-tissue graft sutured in inner aspect of buccal flap, 1 mm apical with respect to mucosal margin, with two horizontal internal mattress sutures.

The connective-tissue graft was harvested from the posterior palate as a free gingival graft and extra-orally de-epithelialized with a surgical blade, with attention paid to also removing glandular and fatty tissue [18]. The apico-coronal dimension of the connective-tissue graft was chosen by taking into consideration two reference points: the gingival margin of the contralateral tooth and the buccal bone crest. The graft should be sutured 1 mm coronal with respect to the ideal position of the mucosal margin, and it must be 2–3 mm apical with respect to the bone crest. The mesial distal dimension of the graft was extended to include the width of both papillae. This facilitated graft suturing and made peri-implant papilla growth in thickness and height possible. The graft was sutured in the inner aspect of the buccal flap 1 mm apical with respect to the mucosal margin with two horizontal internal mattress sutures (7/0 PGA thread; 8 mm needle) (Figure 9).

After the de-epithelialization of the anatomical papillae involved in the surgical area, the flap was sutured coronally. Flap closure started from the periphery and was achieved with two sling sutures suspended around the cingulum of the teeth adjacent to the extracted-tooth site.

After completing the peripheral sutures, the central portion of the flap almost reached the desired coronal position. The soft tissues buccal with respect to the extracted-tooth site should be sutured last in order to minimize the tension in that area.

**Figure 10:**
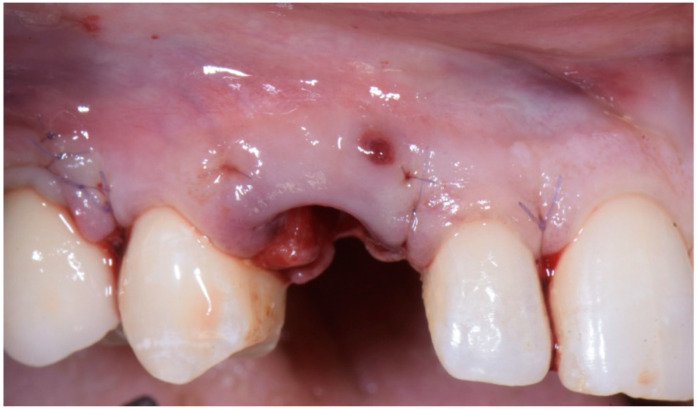
Initial suture of the weakest papilla (the mesial one) before placing the provisional crown, obtaining an ideal adaptation of the surgical papilla on top of the smaller anatomical papilla.

**Figure 11:**
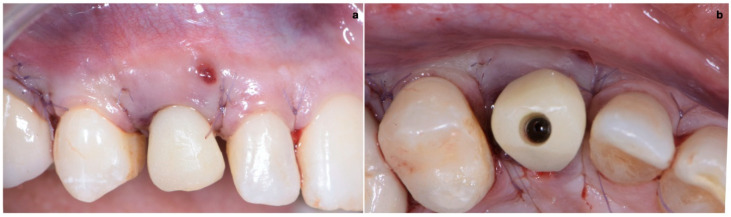
(**a**,**b**) Suture of the second surgical papilla (the distal one) onto the larger anatomical papilla with a simple interrupted suture, after screwing the provisional.

The closure of the surgical papilla over the anatomical papilla was performed with simple interrupted sutures (7/0 thread; 8 mm needle). First, we sutured the weakest papilla (in this case, the mesial one) before placing the provisional crown (Figure 10); this simplified suturing and made it possible to obtain an ideal adaptation of the surgical papilla on top of the smaller anatomical papilla. After screwing the provisional, it was time to suture the second surgical papilla (the distal one) onto the larger anatomical papilla with another simple interrupted suture (Figure 11a,b). 

**Figure 12:**
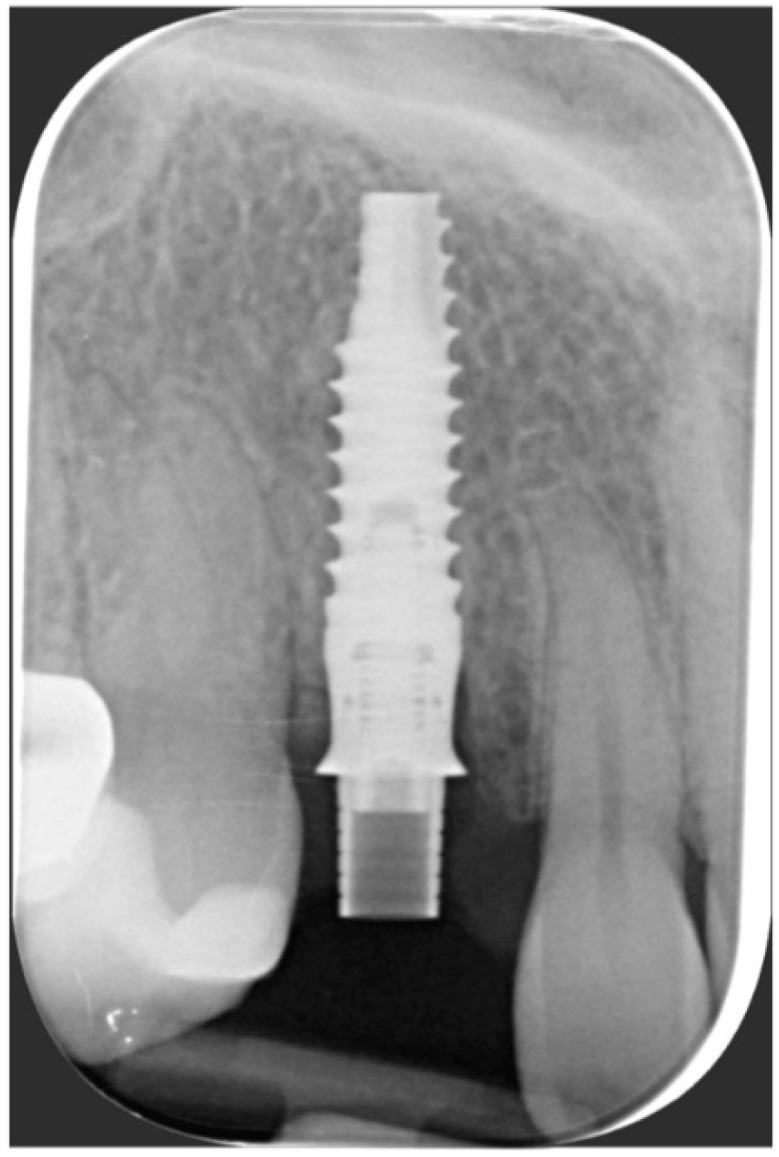
Radiographic evaluation following implant placement.

After the complete closure of the papillae, a final sling suture suspended around the cingulum of the implant-supported crown (6/0 PGA thread; 11 mm needle) was performed to obtain a tight adaptation of the flap’s keratinized tissue into the convex, smooth surface of the provisional, a prerequisite for the stability of the surgical wound and for minimizing the risk of flap shrinkage. The absence of blood seeping from the margins is a sign of surgical wound stability, and this can minimize the contraction of the coronally advanced flap. At the end of surgery, an X-ray scan was performed (Figure 12).

### 2.3. Provisional Restoration

**Figure 13:**
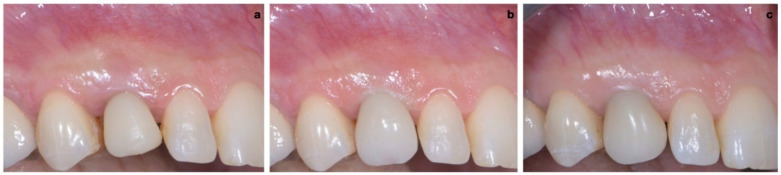
Maturation of soft tissue at 1 month (**a**), 3 months (**b**) and 6 months (**c**) after surgery.

**Figure 14:**
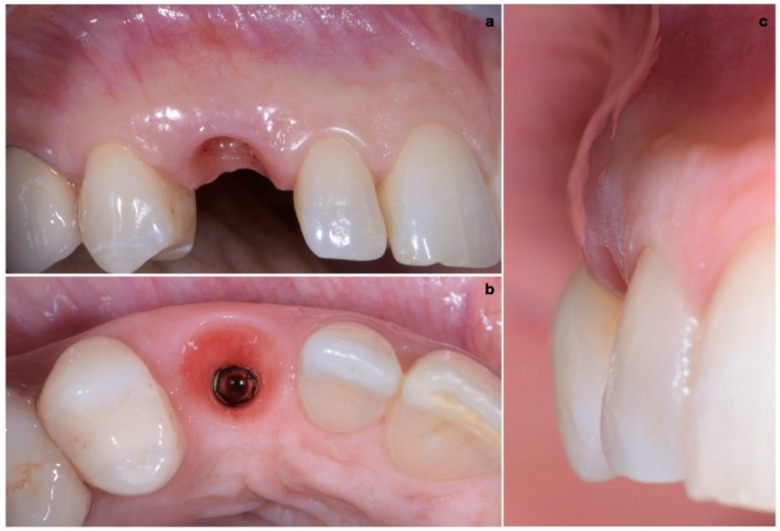
(**a**–**c**) Conclusion of the conditioning phase.

As shown in Figure 13, the maturation of soft tissue at 1 month (a), 3 months (b) and 6 months (c) after surgery was evident. The peri-implant soft tissues were conditioned with the provisional crown until the shape and position for the mucosal scallop to resemble the gingival margin of the adjacent corresponding tooth and the progressive growth of peri-implant papillae were obtained [19]. In this phase, the patient was called every 2 weeks for us to check the provisional restoration. At the end of the conditioning phase, it was possible to place the definitive, screw-retained restoration (Figure 14a–c).

### 2.4. Final Result

**Figure 15:**
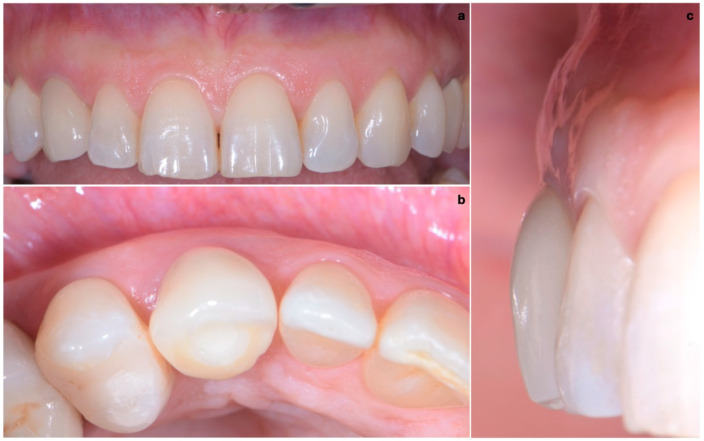
(**a**–**c**) One year after final restoration placement, the clinical outcome remained stable and fully satisfied the patient’s esthetic demands.

**Figure 16:**
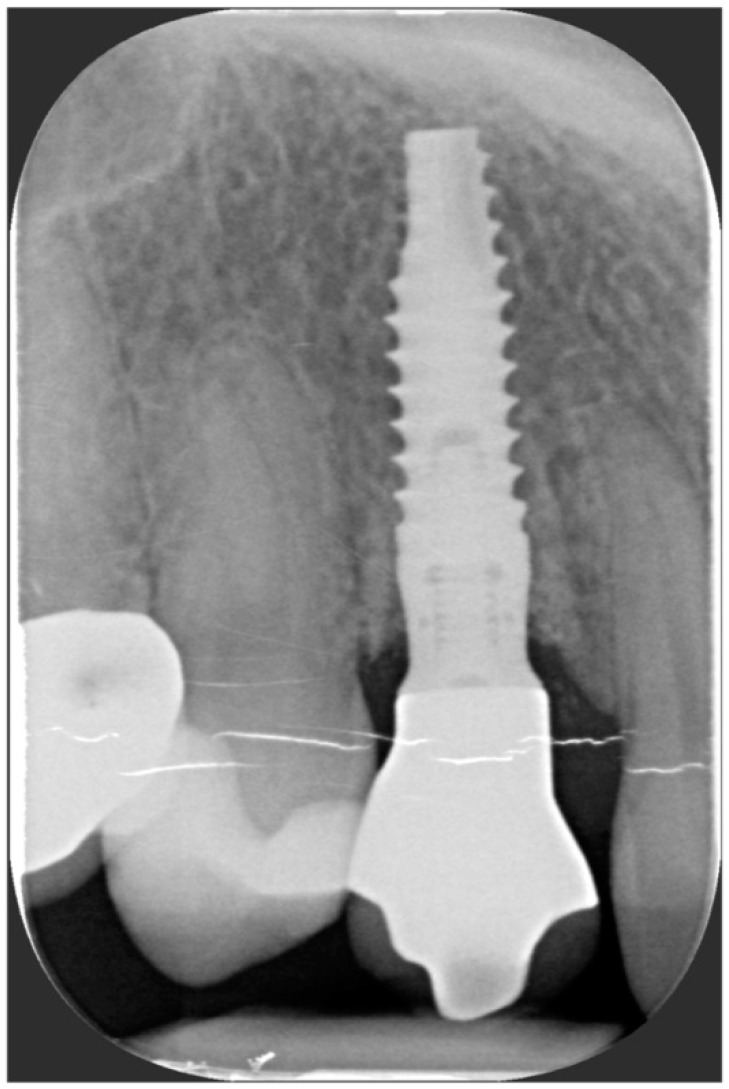
Marginal bone loss evaluated with periodical X-ray scans was minimal.

One year after final restoration placement, the clinical outcome remained stable and fully satisfied the patient’s esthetic demands (Figure 15a–c). The soft tissues were morphologically and dimensionally stable without any signs of inflammation, and the marginal bone loss evaluated with periodical X-ray scans was minimal (Figure 16).

## 3. Discussion

A novel muco-gingival approach for immediate implant placement (MIIP) was adopted in the present study to simultaneously obtain soft- and hard-tissue augmentation. 

The described case, due to the thin phenotype, the presence of a vestibular bone dehiscence and a residual wall thickness <1 mm, represents a contraindication for IIP [16], and traditionally, early implant placement with soft-tissue healing (Type 2) or delayed with soft- and hard-tissue healing would be recommended to prevent the risk of mid-buccal soft-tissue dehiscence.

Recently, a paper evaluating the esthetic outcomes of immediate implant placement with immediate provisionalization in the anterior maxilla with buccal dehiscence [20] concluded that a missing buccal lamellar bone appeared to be no contraindication for immediate implant placement and immediate restoration. The same conclusions were described in a case series by Tirone et al. [21] in even more challenging conditions of IIP in post-extraction sockets with more than 50% impairment of the buccal bone plate. 

Moreover, a recent systematic review concluded that when an elevated risk of mid-facial recession is expected in the aesthetic zone following IIP (thin gingival biotype, <0.5 mm buccal bone thickness), the adjunctive use of a CTG contributes to mid-facial soft-tissue stability [22].

Bearing in mind these new concepts, the presented muco-gingival approach aimed to overcome the factors traditionally considered as contraindications for IIP and immediate provisionalization.

IIP is usually intended in a flapless way, aiming at reducing the surgical trauma and the risk of buccal soft-tissue dehiscence occurrence.

In the MIIP approach, flap elevation is crucial and associated with a variety of advantages.

After raising a flap, tooth extraction is easier and can be performed with less risk of trauma to interproximal soft tissues. Better visibility makes it easier to distinguish between areas in need of bone reconstruction or soft-tissue augmentation and areas that do not need any intervention. 

In the presented case, a small buccal dehiscence (≤3 mm) was treated with a mixture of biomaterial and autologous bone with a resorbable pericardium membrane whose main objective was to give stability to the bone graft in the absence of the buccal plate. Excessive amounts of biomaterial/autologous bone, which could impinge on the supracrestal implant portion, should be removed, leaving the bone graft only at a level where buccal bone reconstruction is predictable before placement of the connective-tissue graft.

Moreover, flap elevation is followed by a coronal advancement that makes it possible to achieve the complete coverage of the most coronal-correct positioning of the connective-tissue graft and compensate for flap shrinkage.

The most coronal placement of the graft (1 mm coronal with respect to the ideal position of the mucosal margin) was chosen to compensate for future tissue contraction and to have, once the tissues healed, buccal soft-tissue margin that was coronally positioned in relation to the gingival margin of the contralateral tooth and that could be prosthetically conditioned in order to obtain a mucosal margin of the implant crown that was at the same height and had the same morphology of the gingival margin of the homologous tooth. The graft was sutured to the flap’s internal surface at the base of the surgical papillae in a paramarginal position with internal horizontal mattress sutures, keeping the anatomical de-epithelialized papillae free from any interference, making first intention wound healing with the surgical papillae of the coronally advanced flap possible. Furthermore, this graft stabilization allowed us to extend the mesial distal dimension of the graft that could be extended to include the width of both papillae. This allowed us to increase the buccal lingual thickness of the interproximal soft tissue, which prevented early papilla shrinkage and furthered papilla growth in height during the conditioning phase with the temporary crown. Suturing the graft to the flap’s internal surface does not preclude the graft from acting as a membrane for the stabilization of biomaterial/autologous bone whenever said bone graft is not completely protected by the alveolar bone due to the presence of bone dehiscence. 

Guided implant placement plays another fundamental role in MIIP. Firstly, it limits possible error in implant positioning, which represents the main predisposing factor for soft-tissue dehiscence occurrence [23,24]; secondly, it makes maximum usage of the bone apical and lateral with respect to the root to be extracted possible; thirdly, it minimizes the surgical trauma and thus the associated bone loss. All of these aspects increase the chances of reaching primary stability suitable for immediate implant provisionalization, a key element for the attainment of the best esthetic result. 

Moreover, a provisional crown rather precise provided by technicians based on pre-surgical planning can be easily relined and be ready at the end of surgery before suturing the flap, adapting it to the convex buccal surface of the temporary crown, thus allowing flap stability to be obtained.

Finally, it should be taken into consideration that post-extractive implants are the first-choice treatment for patients that require tooth extraction in the esthetic area only when the clinical conditions are suitable for the immediate provisionalization of the implant. In fact, the esthetic advantages are mainly related to the ability of the provisional crown to support the soft tissues, preventing their collapse and shrinkage. In the presence of tooth extraction for periodontal reasons, this approach should not be taken into consideration for two main reasons: firstly, periodontal disease must be treated prior to implant tooth replacement, and secondly, even in the rare case of a localized form of periodontitis that affects only one tooth (sporadic loss of attachment), if the extent of bone loss excludes the possibility of performing regenerative periodontal therapy, this is a significant reason not to place a post-extractive implant with immediate provisionalization [25].

## 4. Conclusions

Within the limitations of this case report, the proposed MIIP, immediately temporized through combining CTG application and buccal bone regeneration, shows the possibility of obtaining 1-year-follow-up implant success, stable bone level, good esthetic results and high patient satisfaction. 

Controlled randomized clinical trials with longer follow-ups are advocated to confirm the efficacy of this approach.
